# Association between Altitude and the Framingham Risk Score: A Cross-Sectional Study in the Peruvian Adult Population

**DOI:** 10.3390/ijerph19073838

**Published:** 2022-03-24

**Authors:** Akram Hernández-Vásquez, Rodrigo Vargas-Fernández, Manuel Chacón-Diaz

**Affiliations:** 1 Centro de Excelencia en Investigaciones Económicas y Sociales en Salud, Vicerrectorado de Investigación, Universidad San Ignacio de Loyola, Lima 15024, Peru; 2 Facultad de Ciencias de la Salud, Universidad Científica del Sur, Lima 150142, Peru; jvargasf@cientifica.edu.pe; 3 Instituto Nacional Cardiovascular, EsSalud, Lima 15072, Peru; manuelchaconcardio@gmail.com

**Keywords:** cardiovascular diseases, altitude, cardiovascular risk factors, cross-sectional studies, Peru

## Abstract

To determine the association between altitude and the Framingham risk score in the Peruvian population, we performed a cross-sectional analytical study of data collected by the 2017–2018 Food and Nutrition Surveillance by Life Stages survey. The outcome of this study was the Framingham 10-year cardiovascular disease event risk prediction, which is composed of six modifiable and non-modifiable coronary risk factors. A generalized linear model (GLM) of the gamma family and log link function was used to report the crude and adjusted β coefficients. Several sensitivity analyses were performed to assess the association of interest. Data from a total of 833 surveyed participants were included. After adjusting for educational level, poverty level, alcohol consumption, physical activity level, the presence of any limitation, obesity, and area of residence, it was observed that altitude ≥ 2500 m above sea level (β = −0.42 [95% CI: −0.69 to −0.16]) was negatively and significantly associated with a decrease in the Framingham 10-year risk score. High altitude was significantly and negatively associated with Framingham 10-year risk scores. Our results will allow prevention strategies considering modifiable risk factors to avoid the development of cardiovascular diseases, especially in people living at low altitudes.

## 1. Introduction

Cardiovascular disease (CVD) is a global health problem that has seen a nearly two-fold increase in the prevalence of cases over the last two decades [1]. Although Sustainable Development Goal 3.4 seeks to reduce premature mortality from non-communicable diseases by one-third by 2030 [2], more than 500 million people are living with CVD around the world, and more than 18 million people died from these diseases in 2019 [1]. In addition, CVD has generated a significant increase in disability-adjusted life years, with years lived with disability having increased by more than 16 million between 1990 and 2019 [1]. This increase in the prevalence and disease burden of CVD is attributed to changes in population density around the world, with the aging and growing population playing an important role in the development of CVD [3].

The highest rates of CVD mortality and disease burden are observed in low- and middle-income countries (LMICs), in which more than 80% of CVD deaths occur, and almost 40% are premature [4]. These figures reflect the burden of major modifiable risk factors in LMICs, such as high systolic blood pressure (SBP), limited use of antihypertensive medication, smoking, diabetes, dyslipidemia (low levels of high-density lipoprotein, high levels of low-density lipoprotein, and high levels of total cholesterol), obesity, and low physical activity that have large variations between and within countries [5,6,7]. However, most epidemiological studies conducted in LMIC have focused on the prevalence, burden of disease, mortality of CVD, and its risk factors in the general population [8], without considering environmental determinants such as temperature, altitude, climate, and pollution, which are key risk factors related to CVD [9].

According to the biomedical literature, altitude is a factor associated with CVD and has an inverse association with cardiovascular risk factors [10,11,12,13,14] and mortality associated with CVD [15]; however, the exposure time necessary to obtain a cardioprotective effect is not clear. In this regard, long-term exposure to hypobaric hypoxia (in high altitude regions) could be associated with a lower incidence of obesity, arterial hypertension, metabolic syndrome, and diabetes in individuals due to complex interactions between behavioral and environmental conditions [10,11,12,13]. In Peru (one of the countries belonging to the Andean region of South America), high altitude regions are associated with rural areas and are mostly located at 2500 m above sea level (masl) [16]. Despite the fact that people residing in these regions of Peru have a low prevalence of cardiovascular risk factors [17], it is difficult to establish cardiovascular risk in these regions, mainly because of the scarcity of epidemiological studies.

One way to objectify the level of risk of individuals for the development of CVD in the next 10 years or over a lifetime is the use of the Framingham risk score (FRS) [18], which estimates the actual risk level based on the Framingham Heart Study. The FRS is a common and widely used tool in several studies around the world [19] and has six modifiable and non-modifiable coronary risk factors (age, sex, total cholesterol level, high-density lipoprotein level, smoking, and SBP) [20]. This risk score allows health professionals and patients to generate lifestyle modifications, preventive medical treatment, and medical education in populations at increased risk of CVD.

Despite the availability of tools such as the FRS that can establish cardiovascular risk in specific populations, there is still little scientific evidence on the association between the level of cardiovascular risk and altitude of residence in Peru. Therefore, the aim of the present study was to determine the association between altitude and the FRS in the Peruvian population.

## 2. Materials and Methods

### 2.1. Study Design and Data Source

We performed a cross-sectional analytical study of data collected by the 2017–2018 Food and Nutrition Surveillance by Life Stages survey (VIANEV-acronym in Spanish), which was conducted by the National Food and Nutrition Center of the National Institute of Health of Peru [21]. The 2017–2018 VIANEV survey is a Peruvian survey that included interviews, anthropometric measurements, and biomarker analyses and is representative at the country level of adults aged 18 to 59 years old residing in households [21].

The 2017–2018 VIANEV collects data from three domains: Metropolitan Lima (capital of Peru) and the remaining urban and rural areas through a stratified, multistage, probabilistic, and independent sampling process [21]. The sample selection was conducted in two stages, with the primary sampling unit being a sample of randomly selected clusters and the secondary sampling unit being a sample of randomly selected households with adults aged 18–59 years, with a fasting period of at least 9 to 12 h. The sampling frame of the VIANEV survey consisted of the households that make up the National Household Survey (ENAHO in Spanish) sample, which included 1296 clusters (176 in Metropolitan Lima and Callao, 696 in the remaining urban areas, and 424 in rural areas). The level of inference is at the national, urban, rural, and Metropolitan Lima (capital of Peru) level. Further details of the methodology can be found in the Technical Report of the 2017–2018 VIANEV survey and previous studies [21,22,23].

**Figure 1:**
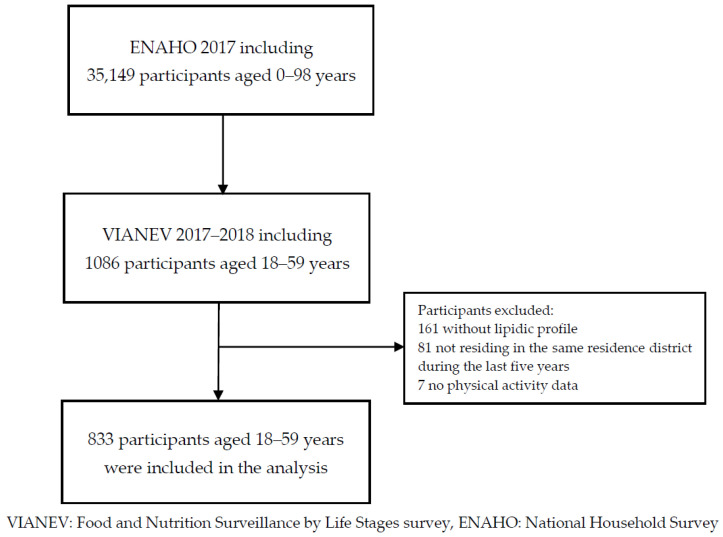
Flow chart of participants included in the study analysis.

The survey included a total of 1086 subjects [21], and 833 subjects were included in the present study according to the inclusion criteria (Figure 1): (1) subjects aged 18 to 59 years; (2) complete data of the FRS and variables of interest; and (3) people residing in the same residence district during the last five years. The exclusion criteria were: (1) non-residents of the household and (2) pregnant women.

### 2.2. Variables

#### 2.2.1. Outcome Measure

The primary outcome of this study was the Framingham 10-year cardiovascular disease event risk prediction [18]. The Framingham score is a gender-specific algorithm that includes: (a) age in years, (b) SBP in mmHg, (c) use of anti-hypertensive treatment, (d) current smoking status, (e) diabetes (glucose ≥ 126 mg/dL), (f) high-density lipoprotein level in mg/dL, and (g) cholesterol level in mg/dL [18]. For the present study, individuals were classified according to the Framingham 10-year cardiovascular disease event risk prediction using the command *framingham* with Stata version 14 (StataCorp, College Station, TX, USA) [24], and the score obtained was included in the analysis as a numerical variable.

#### 2.2.2. Exposure

Exposure was defined as altitude, measured in masl (low: ≤500/medium >500 and <2500/high ≥2500), which was measured for every household conglomerate of the surveyed individual. The cut-off points used were based on the definition of high altitude (≥2500) of Barry and Poland [25], and the population distribution by altitude of the Cohen and Small classification [26].

#### 2.2.3. Covariates

We used the multilevel influences on young adult cardiovascular health conceptual framework of the National Heart, Lung, and Blood Institute to determine potential confounders [27]. We included a group of covariates representing demographic, socioeconomic, and exposure to health risk factors based on previous studies [10,28,29]. The co-variables considered in the analysis were: educational level (categorized as up to primary, secondary, and higher education), poverty level (categorized as non-poor or poor), area of residence (categorized as rural and urban), alcohol intake in the last 30 days (categorized as no or yes), level of physical activity according to the short version of the International Physical Activity Questionnaire (categorized as moderate/high or low), self-reporting of the presence of any physical, psychological, or cognitive limitation (categorized as no or yes), and obesity, which was constructed based on the body mass index (BMI). The BMI was calculated according to the Quetelet index as weight (kg) divided by height squared (m^2^), and the variable obesity was dichotomized as yes, when the BMI value was 30 or more and no, when the BMI was less than 30.

### 2.3. Statistical Analysis

All analyses were performed with Stata version 14 (StataCorp, College Station, TX, USA), and a *p* < 0.05 was considered statistically significant. The 2017–2018 VIANEV survey databases were merged with the ENAHO 2017 databases that are freely available on the National Institute of Statistics and Informatics (INEI-acronym in Spanish) website (http://iinei.inei.gob.pe/microdatos/) according to a previous study [23]. The analysis was weighted to account for the individual survey sample design and cluster size.

Description of the study sample was conducted using frequencies and weighted proportions and means with standard deviation (SD) or median interquartile range according to the type of variable. Weighted proportions, together with the 95% confidence interval (95% CI) and means, together with their 95% CI for numerical variables, were used to describe exposure and the FRS or its components. The Rao–Scott Chi-square statistical test and the F-test were performed to compare differences in the outcome variable across altitude. To evaluate the association between altitude and FRS, univariate, and multivariable generalized linear models (GLM) of the gamma family (due to the skewed positive data) and logarithmic link function were performed to report the crude and adjusted β coefficients with their respective 95% CI. The variance inflation factor (VIF) was used to check multicollinearity.

We performed several sensitivity analyses. Firstly, we ran the multivariable GLM incorporating the potential of confounders such as sex and age to the model. Secondly, we examined the association between altitude as a numerical variable and the FRS from a multivariable GLM, and the marginal effects were calculated and plotted together with their 95% CI.

### 2.4. Ethical Considerations

The National Institute of Health provided the anonymized database of the 2017–2018 VIANEV survey data after request for access to public information (https://web.ins.gob.pe/es/transparencia/solicitud-de-acceso-a-la-informacion-publica). The ENAHO 2017 microdata were obtained from the INEI website (http://iinei.inei.gob.pe/microdatos/). Ethical approval was not required for this research due to the public and anonymous nature of the data used.

## 3. Results

Data from a total of 833 surveyed participants, with a mean age of 38.2 (SD: 11.9) years and a median Framingham 10-year risk score of 2.1 points (interquartile range: 0.8–5.5) were included. Most participants were female (57.9%), with a higher educational level (41.6%), had consumed alcohol in the last month (53.4%), had a low level of physical activity (60.5%), and resided in an urban area (80.5%), while 14.4% were poor, 2.0% presented some limitation, and 15.6% resided at an altitude of 2500 masl or more (Table 1).

Regarding the Framingham 10-year risk score according to altitude levels, it was found that participants residing at an altitude of ≤500 masl had a mean score of 4.9 points, whereas the mean scores in those at an altitude from >500 masl to <2500 masl, and ≥2500 masl were 4.5 and 3.4, respectively (Table 2). In relation to the coronary risk factors that make up the Framingham 10-year risk score, it was found that participants residing at an altitude of ≤500 masl had a mean SBP of 108.7 mmHg, and 12.6% had diabetes (fasting glucose ≥ 126 mg/dL), while participants residing at an altitude of ≥2500 masl had a mean SBP of 104.3 mmHg, and 3.1% had a diagnosis of diabetes (Table 2).

In the analysis adjusted for educational level, poverty level, alcohol consumption, physical activity level, the presence of any limitation, obesity, and area of residence, it was observed that altitude ≥ 2500 masl (β = −0.42 [95% CI: −0.69 to −0.16]) was negatively and significantly associated with a lower Framingham 10-year risk score (Table 3).

**Figure 2:**
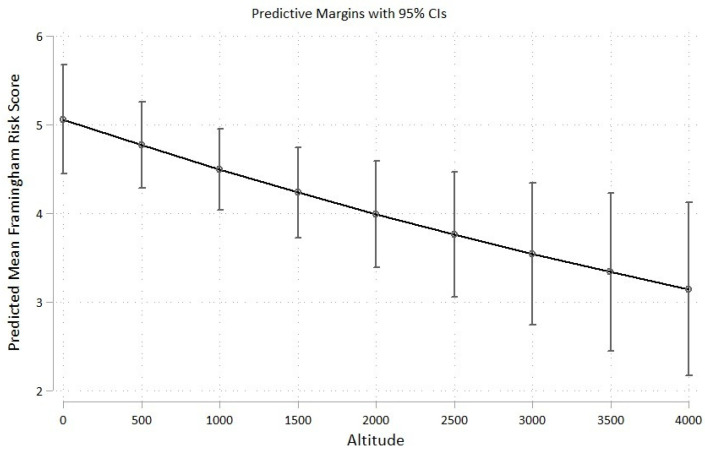
Predictive margins for the relationship between altitude as a numerical variable and the Framingham risk score.

Sensitivity analyses showed that the incorporation of sex and age as potential confounders (both components of the Framingham 10-year risk score) did not have a great impact on the final estimates (Table 4). In addition, incorporating altitude as a numerical variable did not change the direction of the association estimate and showed a nonlinear inverse relationship (Table 5 and Figure 2).

## 4. Discussion

The present study sought to determine the association between altitude and the FRS in the Peruvian adult population. We found that altitude ≥ 2500 masl was significantly and negatively associated with the Framingham 10-year risk score. Regarding the components of the coronary risk factors that make up the Framingham 10-year risk score, a lower mean SBP, and a lower diagnosis of diabetes were found in persons residing at an altitude ≥ 2500 masl compared to residents at an altitude ≤ 500 masl.

An altitude of residence ≥ 2500 masl was negatively and significantly associated with decreased Framingham 10-year risk scores. This finding is in agreement with that reported in studies conducted in high-altitude regions of Switzerland [30], Austria [31], Greece [32], the United States [33], and Ecuador [34], in which it was found that living in a high-altitude region has a protective effect on cardiovascular mortality and the incidence of cardiovascular events, especially coronary heart disease and stroke. From animal models, it is postulated that altitude has a cardioprotective effect due to biological mechanisms associated with hypobaric hypoxia. Chronic hypoxia occurring in residents of high-altitude regions generates a moderate production of reactive oxygen species that activates the expression of antioxidant and anti-inflammatory genes that increase cardiomyocyte resistance to hypoxia [35]. In addition, cardiomyocytes synthesize proteins such as hypoxia-inducible factor, which induces the expression of multiple genes with cardioprotective properties, including stimulation of nitric oxide synthesis, increased erythropoietin production, angiogenesis, increased mitochondrial energy production, and other antioxidant functions [36,37,38].

On the other hand, residents of high-altitude regions have their own characteristics that differ from people living in low-altitude regions, just as there are climatic and geographic characteristics of high-altitude regions. In high-altitude regions, it is observed that people show greater physical activity related to manual agricultural and livestock work and transport on foot, as well as low fat consumption and limited consumption of processed foods, all of which reduce the prevalence of coronary risk factors [39,40]. These regions have a low level of pollution and greater exposure to ultraviolet rays, which increases vitamin D synthesis [41]. In animal models and in humans, it is postulated that vitamin D could have cardioprotective characteristics; however, its protective effect is controversial due to the contradictory results of epidemiological studies [42]. Thus, residents of high-altitude regions have biological and sociodemographic characteristics, as well as characteristics specific to these regions, that reduce the risk of cardiovascular events in individuals and promote adequate cardiovascular health.

Regarding the components of the Framingham 10-year risk score, differences were observed between residents of an altitude ≥ 2500, particularly in the mean SBP, and the diagnosis of diabetes mellitus, showing that lower levels of these two components were observed in individuals residing at an altitude ≥ 2500 masl. This result is similar to that reported in studies conducted in high-altitude regions of Austria [43], Peru [11], Nepal [44], and China [12], in which an inverse association was found between arterial hypertension and diabetes mellitus, and altitude. This finding could be attributed to the fact that people residing in high-altitude regions develop protective mechanisms against hypobaric hypoxia from birth and have a lower tendency of developing hypertension and having conventional blood pressure measurements that may underestimate the prevalence of hypertension in this population [12,45]. In addition, this population experiences low levels of urbanization, low income, and healthier lifestyles (more physical activity and lower fat consumption) compared to people living in low-altitude regions [46,47,48], thereby producing a lower prevalence of coronary risk factors.

Our findings may improve actions in clinical practice and public health policies, especially considering that the results suggest that altitude is a protective factor for cardiovascular events. In clinical practice, this finding will allow physicians or health personnel working in low-altitude regions to conduct more screening campaigns for CVD and for risk factors that may increase the risk of these diseases. On the other hand, Peruvian governmental programs should consider specific strategies of awareness and education on healthy lifestyles in people who present a high prevalence of coronary risk factors and live in low-altitude regions. Thus, the ultimate goal of the programs implemented should be to prevent the development of CVD, disease burden, and mortality.

Among the limitations of the study is the impossibility of establishing causality in the association presented due to the lack of temporality in the variables measured. The VIANEV database was used for the present study; however, this survey was not intended to evaluate the association between altitude and FRS. As this was a secondary data study, there may be recall bias, errors in recording information, and social desirability bias, especially with information on habits that are harmful to health. Additionally, people with comorbidities may have been included in the study since it is based on population-based data that does not collect specific information on participants’ pre-existing comorbidities. On the other hand, future research that takes into account time of residence is needed to determine whether the results of the present study are similar in individuals who are natives or permanent residents of high-altitude regions. Despite these limitations, the 2017–2018 VIANEV is a population-based, multistage survey that was conducted by previously trained personnel to ensure adequate data collection. Although evidence assessing the validity of the FRS in the Latin American population is limited, and without proper calibration may overestimate CVD risk in individuals, this study allows an approximation of CVD risk in a population residing in high-altitude regions and serves as a basis for future research.

## 5. Conclusions

In conclusion, we found that altitude was significantly and negatively associated with the Framingham 10-year risk scores. Likewise, it was observed that the mean SBP and diagnosis of diabetes mellitus had lower values at an altitude ≥ 2500 masl compared to low-altitude regions. The findings of the present study will allow the design of prevention strategies considering modifiable risk factors to avoid the development of cardiovascular diseases and reduce the burden of disease and mortality in people living at low altitudes.

## Figures and Tables

**Table 1:** Characteristics of the participants included in the study.

Characteristics	Absolute Frequency (*n* = 833)	Weighted Proportion *
Age (years)		
Mean (SD)		38.2 (11.9)
Sex		
Women	482	57.9 (54.3–61.4)
Men	351	42.1 (38.6–45.7)
Educational level		
Up to primary	202	18.2 (15.6–21.1)
Secondary	347	40.2 (35.9–44.6)
Higher education	284	41.6 (37.1–46.2)
Poverty		
Non-poor	692	85.6 (82.3–88.4)
Poor	141	14.4 (11.6–17.7)
Area of residence		
Rural	280	19.5 (17.6–21.6)
Urban	553	80.5 (78.4–82.4)
Alcohol consumption in the last month		
No	425	46.6 (42.6–50.6)
Yes	408	53.4 (49.4–57.4)
Level of physical activity		
Moderate/High	353	39.5 (35.6–43.6)
Low	480	60.5 (56.4–64.4)
No	811	98.0 (96.7–98.7)
Yes	22	2.0 (1.3–3.3)
Obesity		
No	617	73.2 (69.4–76.7)
Yes	216	26.8 (23.3–30.6)
Altitude of residence (masl)		
≤500	577	73.1 (68.8–77.0)
>500 and <2500	118	11.3 (8.6–14.9)
≥2500	138	15.6 (12.3–19.5)
Framingham 10-year risk score		
Mean (SD)		4.6 (6.5)
Median (IQR)		2.1 (0.8–5.5)

* Estimates include the weights and 2017–2018 VIANEV sample specifications. SD: standard deviation; IQR: interquartile range; masl: meters above sea level.

**Table 2:** Characteristics of the participants according to the Framingham criteria and altitude levels.

Characteristics		Altitude			
	Overall	≤500 masl (*n* = 577)	>500 and <2500 masl (*n* = 118)	≥2500 masl (*n* = 138)	
	% or Mean (95% CI)	% or Mean (95% CI)	% or Mean (95% CI)	% or Mean (95% CI)	*p*-Value *
Total score	4.6 (4.1–5.1)	4.9 (4.3–5.5)	4.5 (2.7–6.3)	3.4 (2.8–4.1)	0.006
Age (years)					
Mean	38.2 (37.3–39.1)	38.1 (37.2–39.1)	38.3 (35.8–40.9)	382 (35.9–40.9)	0.989
Sex					
Women	57.9 (54.3–61.4)	57.9 (53.8–61.9)	59.9 (48.9–70.1)	56.3 (46.3–65.9)	0.888
Men	42.1 (38.6–45.7)	42.1 (38.1–46.2)	40.1 (29.9–51.1)	43.7 (34.1–53.7)	
Systolic blood pressure (mmHg)					
Mean	107.7 (106.6–108.8)	108.7 (107.3–110.0)	105.9 (103.3–108.6)	104.3 (101.8–106.8)	0.006
Treatment for hypertension					
No	97.3 (95.7–98.3)	96.8 (94.7–98.1)	98.2 (94.7–99.4)	98.8 (95.3–99.7)	0.247
Yes	2.7 (1.7–4.3)	3.2 (1.9–5.3)	1.8 (0.6–5.3)	1.2 (0.3–4.7)	
Smoker					
No	86.8 (83.5–89.5)	86.4 (82.4–89.6)	91.3 (80.3–96.4)	85.7 (76.4–91.8)	0.564
Yes	13.2 (10.5–16.5)	13.6 (10.4–17.6)	8.7 (3.6–19.7)	14.3 (8.2–23.6)	
Diabetes **					
No	89.1 (86.4–91.2)	87.4 (84.2–90.0)	89.3 (79.4–94.7)	96.9 (91.6–98.9)	0.019
Yes	10.9 (8.8–13.6)	12.6 (10.0–15.8)	10.7 (5.3–20.6)	3.1 (1.1–8.4)	
Cholesterol (mg/dL)					
Mean	179.3 (174.9–183.7)	178.9 (173.4–184.3)	177.7 (168.3–187.0)	182.5 (171.8–193.2)	0.785
High-density lipoprotein (mg/dL)					
Mean	36.9 (35.9–37.9)	36.6 (35.4–37.8)	38.4 (34.8–41.9)	37.2 (34.6–39.7)	0.629

Estimates include the weights and VIANEV 2017–2018 sample specifications. * The *p*-value was calculated using the Rao–Scott Chi-squared test or F test. ** Fasting glucose ≥ 126 mg/dL. 95% CI: 95% confidence interval; masl: meters above sea level.

**Table 3:** Association between altitude and the Framingham risk score.

Characteristics	Crude Model		Adjusted Model *	
	β (95% CI)	*p*-Value	β (95% CI)	*p*-Value
Altitude of residence (masl)				
≤500				
>500 and <2500	−0.08 (−0.50 to 0.33)	0.691	−0.17 (−0.56 to 0.22)	0.388
≥2500	−0.36 (−0.58 to −0.13)	0.002	−0.42 (−0.69 to −0.16)	0.002

* Adjusted for educational level, poverty, alcohol consumption, level of physical activity, presence of some limitation, obesity, and area of residence. β: beta coefficient. masl: meters above sea level. 95% CI: 95% confidence interval.

**Table 4:** Sensitivity analysis of the association between altitude and the Framingham risk score.

Characteristics	Crude Model		Adjusted Model *	
	β (95% CI)	*p*-Value	β (95% CI)	*p*-Value
Altitude of residence (masl)				
≤500				
>500 and <2500	−0.08 (−0.50 to 0.33)	0.691	−0.07 (−0.32 to 0.18)	0.606
≥2500	−0.36 (−0.58 to −0.13)	0.002	−0.22 (−0.36 to −0.07)	0.004

* Adjusted for age, sex, educational level, poverty, alcohol consumption, level of physical activity, presence of some limitation, obesity, and area of residence. β: beta coefficient. masl: meters above sea level. 95% CI: 95% confidence interval.

**Table 5:** Sensitivity analysis of the association between altitude as a numerical variable and the Framingham risk score.

Characteristics	Crude Model		Adjusted Model *	
	β (95% CI)	*p*-Value	β (95% CI)	*p*-Value
Altitude of residence (masl)				
Altitude in meters was divided by 100	−0.01 (−0.02 to −0.00)	0.008	−0.01 (−0.02 to −0.00)	0.010

* Adjusted for educational level, poverty, alcohol consumption, level of physical activity, presence of some limitation, obesity, and area of residence. β: beta coefficient. masl: meters above sea level. 95% CI: 95% confidence interval.

## Data Availability

Publicly available datasets were analyzed in this study. This data can be found here: http://iinei.inei.gob.pe/microdatos/ and after request for access to public information here: https://web.ins.gob.pe/es/transparencia/solicitud-de-acceso-a-la-informacion-publica.
